# Supplementary figures and images for: The evolution of the type VI secretion system as a disintegration weapon
Source: PLoS Biol. 2020 May 26;18(5):e3000720. doi: 10.1371/journal.pbio.3000720 (PMC7274471; doi:10.1371/journal.pbio.3000720)

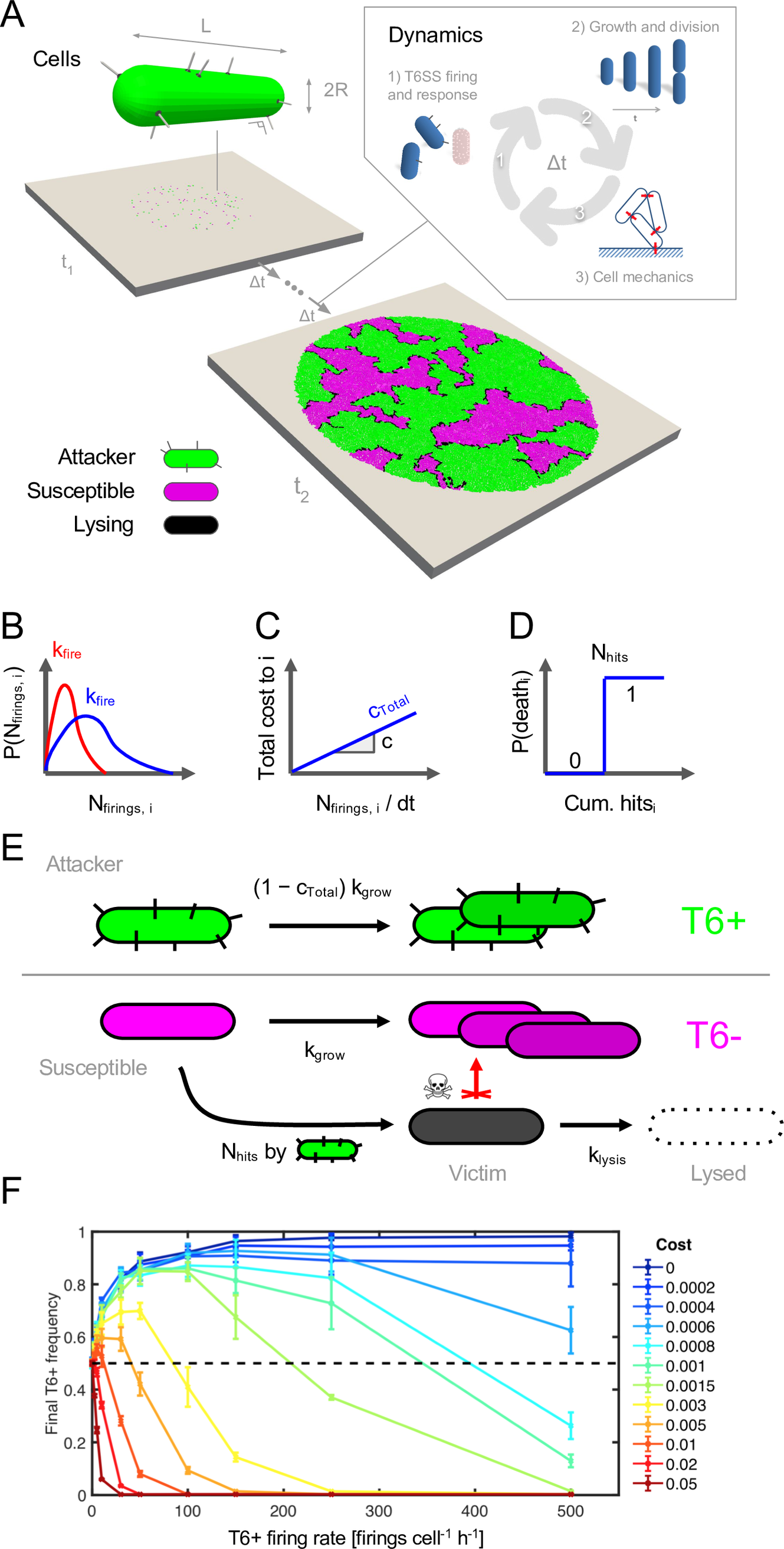

Supplement: S1 Fig — (A) Diagram of 2D competition simulation showing initial (tstart; 100 cells) and final (tend; approximately 10,000 cells) cell configurations. (B) Each simulation timestep, every T6SS+ cell fires Nfirings,i, i times with Nfirings,i drawn independently from a Poisson distribution with mean kfire. (C) Cell’s growth costs are computed from values of Nfirings,i; costs are assumed to scale linearly with a cell’s firing rate. (D) Cells respond to successful translocations with a steplike dose–response curve: once a cell’s cumulative translocation count reaches threshold Nhits, that cell dies and becomes a “Victim”; see (E). Cells of the same genotype are immune to each other’s effectors. (E) Cartoon of cell-based processes summarizing cell-based T6SS firing and response parameters; here, kgrow is the maximum specific growth rate, and klys the lysis rate post-T6SS intoxication. (F) Initial parameter sweep showing final attacker frequencies as a function of T6SS firing rate, for various weapon cost parameters c (legend). Circles and bars denote means and standard deviations; 5 simulation replicates per case. Raw data are available at dx.doi.org/10.6084/m9.figshare.11980491. T6SS, type VI secretion system. (TIF) [file pbio.3000720.s001.tif]

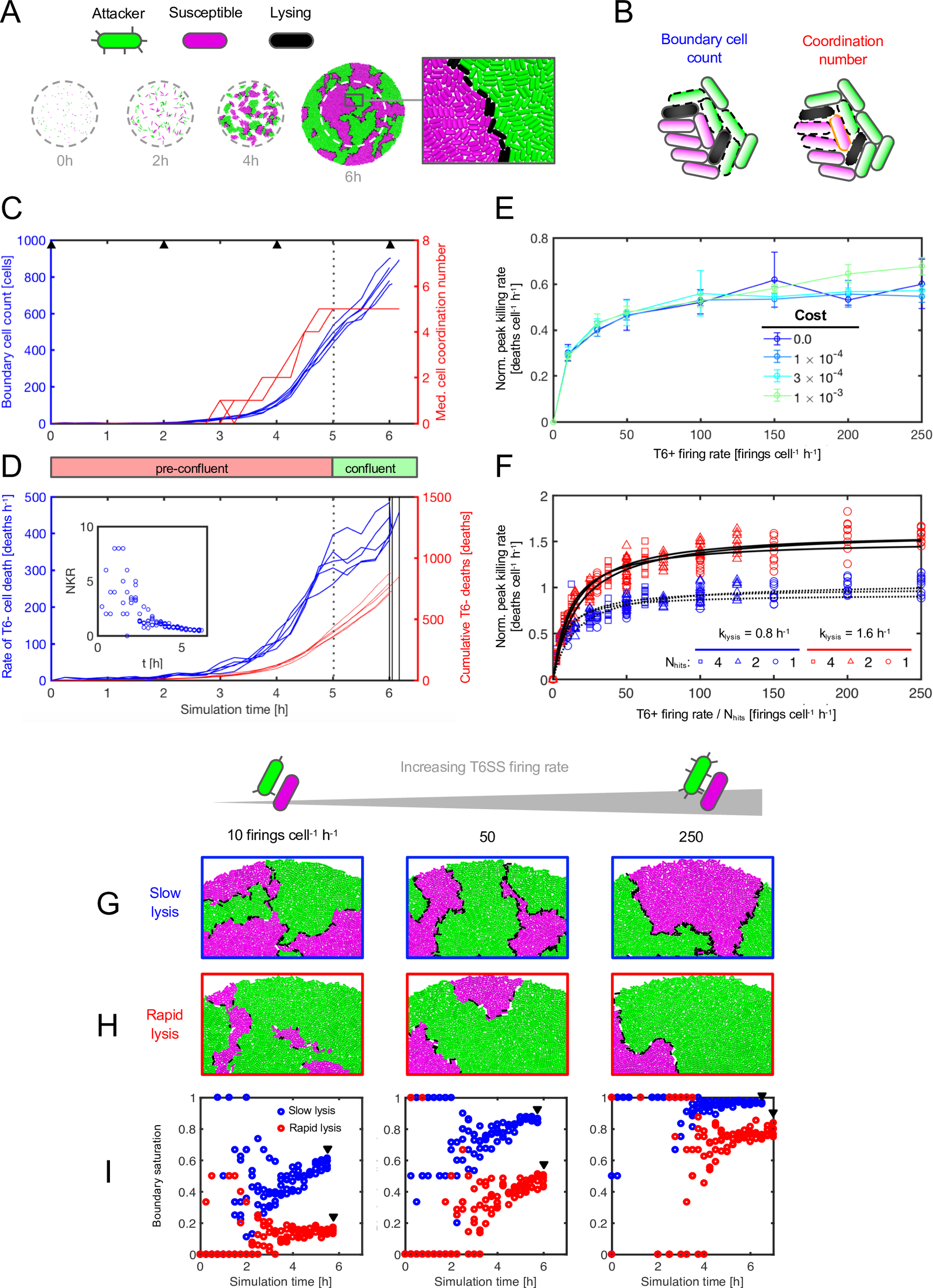

Supplement: S2 Fig — (A) Images of T6SS competition simulations, run as in Fig 1, highlighting a section of interstrain boundary between attacker (T6SS+, green) and susceptible (T6SS−, magenta) cell groups. (B) Diagrams of boundary cell classification (boundary cells shown with dashed outline) and of cell coordination number (5 neighbors of orange focal cell shown with dashed outline). (C) Boundary cell count traces (left axis) show number of T6+ cells in contact with nonkin cells (see B) as function of simulation time. Median cell coordination number (right axis, see B) plateaus to 5 ± 1 cells at confluency, after approximately 5 hours’ growth. Black arrows correspond to the simulation snapshots shown in (A). (D) Absolute kill rates (blue traces, left axis) are measured by counting T6-dependent cell deaths per simulation step and then numerically computing the gradient of cumulative kill count trace (red traces, right axis). These traces are normalized by the number of boundary cells at each corresponding time point to give an NKR per unit interface (inset), which converges to a constant value in confluent colonies. (E) Normalized peak T6– kill rates taken at confluency (the maxima of the raw kill rates; vertical black lines in D), plotted against T6+ firing rate kfire, for different weapon cost parameters c (legend). Circles and bars denote means and standard deviations, respectively. Five simulation replicates per case. (F) Normalized peak T6– kill rates plotted against the ratio kfire / Nhits, for different lysis rates klysis. Symbols denote the value of the lethal hit threshold Nhits used in each simulation (legend). For each of the 6 resulting simulation groups, we found that increasing Nhits was equivalent to proportionally reducing kfire, such that plotting peak kill rates against the ratio kfire / Nhits yielded the same curve for each klysis value. Black lines correspond to Monod curves, fitted for each Nhits value, as a test of their similarity (solid, klysis = 1.6 h-1; dashed [file pbio.3000720.s002.tif]

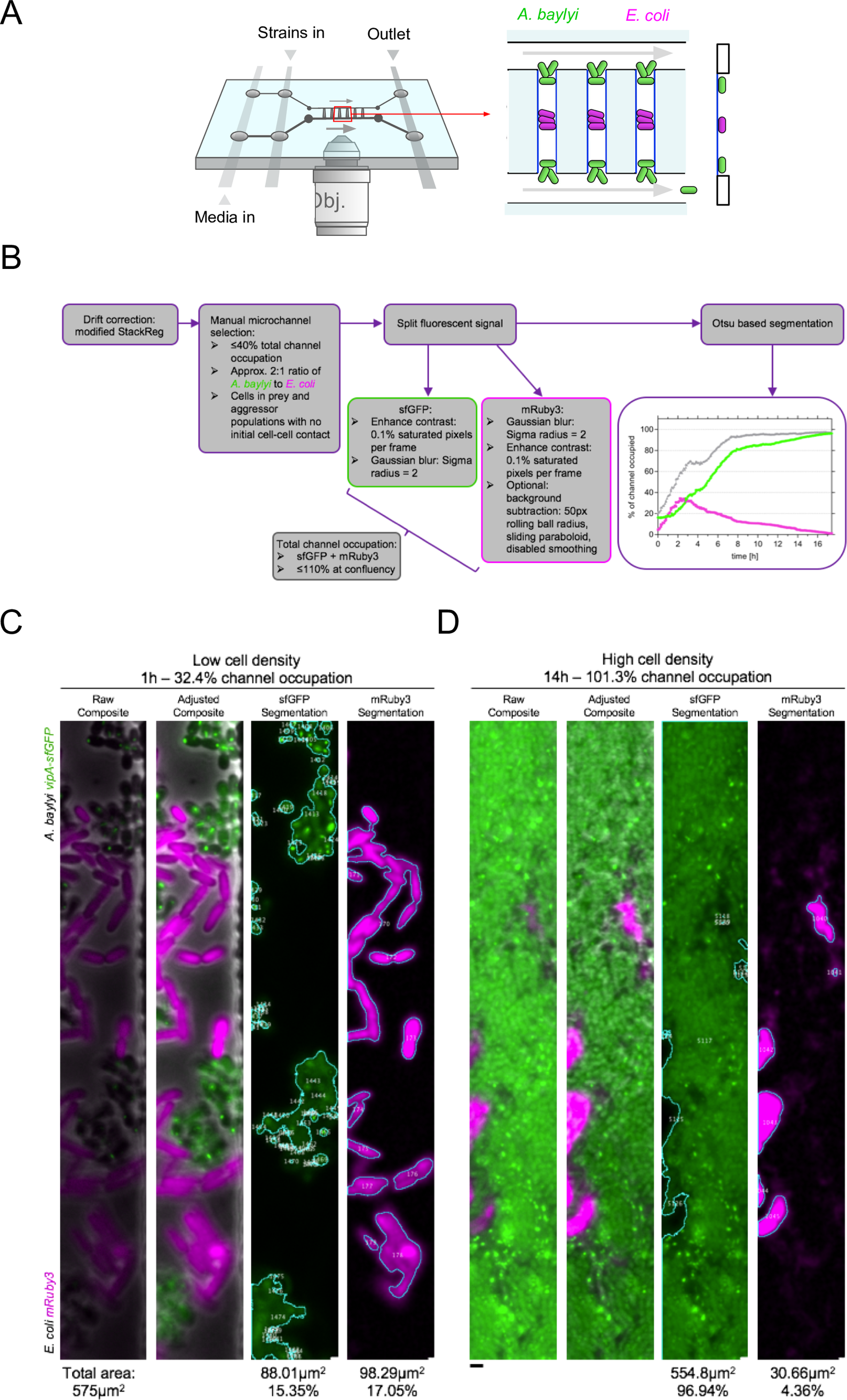

Supplement: S3 Fig — (A) Diagram of microfluidic chip (left), showing inlets, outlets, flow, and observation channels; zoomed section (right) shows observation channels loaded with A. baylyi and T6SS− E. coli. Diagram not to scale. Flow diagram (B) summarizing workflow for image postprocessing and segmentation in Fiji. The graph shown can be found in S5C Fig. (C, D) Examples for image preprocessing (contrast enhancement, blurring, and background subtraction) are shown in the adjusted composite image. GFP and mRuby3 fluorescence signals were used for segmentations (turquois outlines). Examples are provided for low (C) and high (D) chamber occupancies. Scale bar: 2 μm. GFP, green fluorescent protein; mRuby3, monomeric red fluorescent protein; T6SS, type VI secretion system. (TIF) [file pbio.3000720.s003.tif]

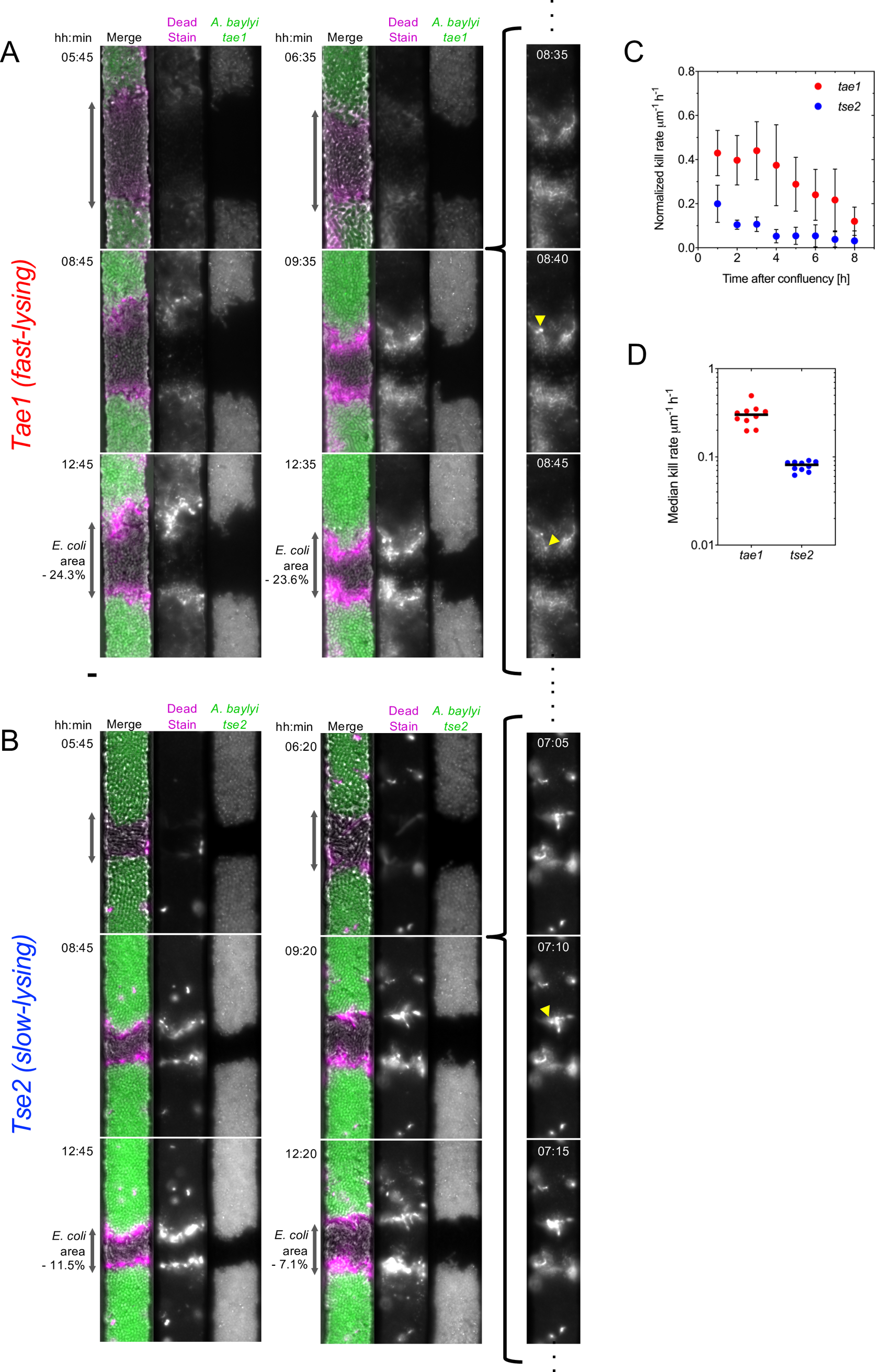

Supplement: S4 Fig — Fast-lysing (A) and slow-lysing (B) single-effector A. baylyi attacker strains (vipA-sfGFP, green) were coincubated with E. coli (unlabeled) victims in the presence of 2 μg ml-1 PI (PI dead stain, magenta), within microfluidic channels. Two additional examples are displayed here, analogous to those shown in Figs 2A and 2B. The percentage reduction in E. coli channel occupancy over 8 hours is indicated, with t = 0 corresponding to the point at which the chamber becomes confluent. To measure the rate of victim cell death, each time-lapse series was split into 1-hour segments (consisting of 12 frames, 5-minute acquisition frame rate) from the moment of confluency, and the number of new PI foci appearing after each hour were counted. Examples of 3 consecutive time points are provided in the far-right column; yellow triangles highlight cell death event. Then, the number of cell death events per hour was normalized to the contact perimeter between E. coli and A. baylyi (based on sfGFP signal at first frame of every 1-hour segment). From this, the victim cell kill rate over time (C) and thence the average victim cell killing rate per hour (D) were determined. This analysis was carried out for 10 separate microchannels for both attacker strains. Scale bars: 2 μm. Raw data are available at dx.doi.org/10.6084/m9.figshare.11980491. PI, propidium iodide; sfGFP, super-folding green fluorescent protein; Tae1, type VI amidase effector 1; Tse2, type VI effector 2; VipA, ClpV-interacting protein A. (TIF) [file pbio.3000720.s004.tif]

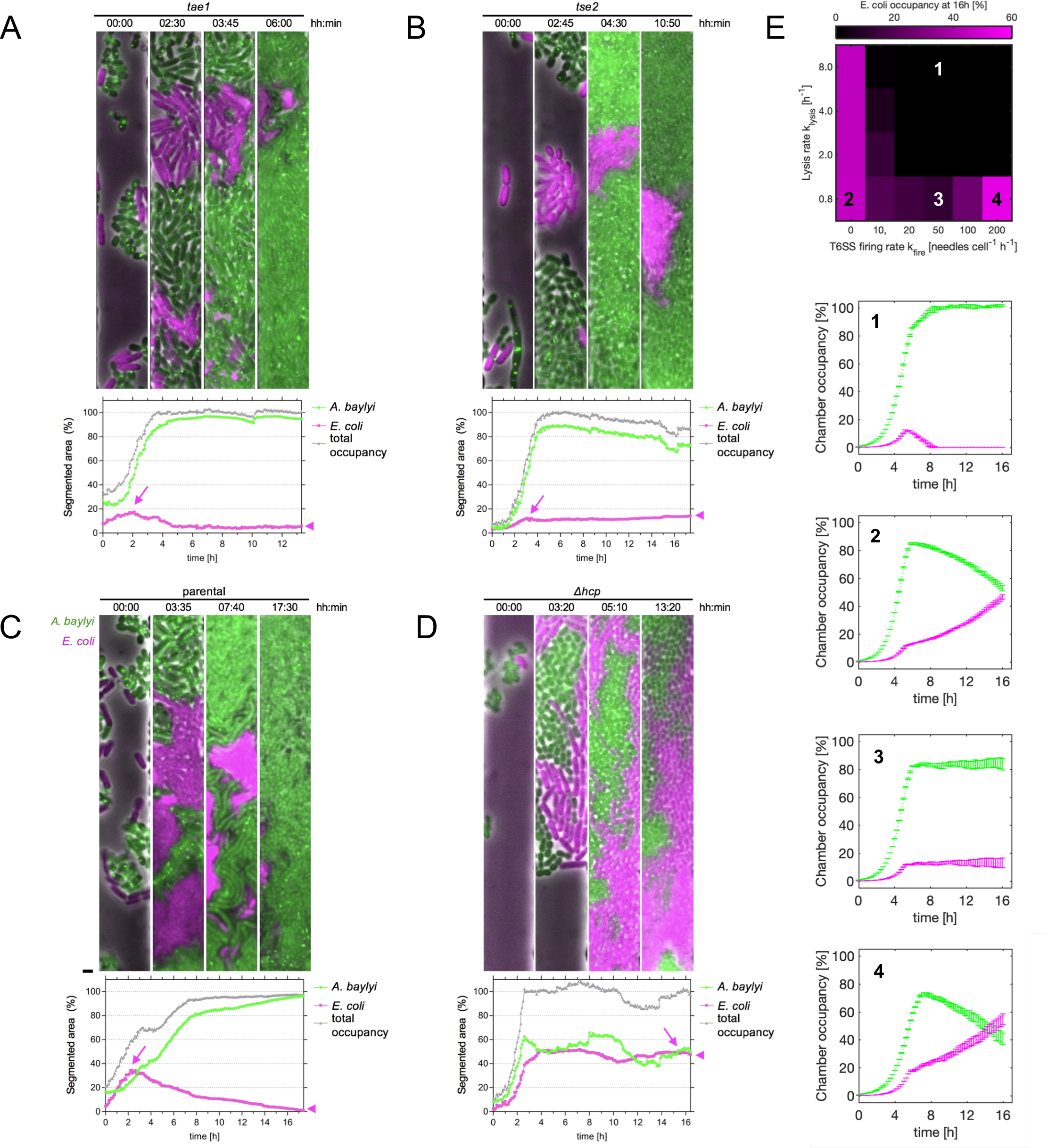

Supplement: S5 Fig — Fluorescence time-lapse series showing microfluidic competition experiments, between E. coli expressing cytosolic mRuby3 (magenta) and A. baylyi expressing vipA-sfGFP (green) armed with different T6SS effectors. (A) A. baylyi secreting Tae1. (B) A. baylyi secreting Tse2. (C) A. baylyi Parental strain. (D) Nonsecreting A. baylyi T6SS− mutant (Δhcp). Below each time series, the corresponding strain area occupancy plot, computed using the procedure described in S3 Fig, is shown. (A, B) are reproduced from Fig 2 but show full channel overview. Additional time-lapse series are shown in S3 Movie (A, B) and S4 Movie (C, D). Scale bar: 2 μm. (E) Color map representing final (= 16 hours) E. coli occupancy in chamber simulations, performed as in Fig 2, for different kfire and klysis parameter values. Four example strain occupancy plots (1–4) are marked and shown below. Legends as in (A–D); dashed lines and bars denote occupancy means and standard deviations, respectively. Five simulation replicates per case. Raw data are available at dx.doi.org/10.6084/m9.figshare.11980491. mRuby3, monomeric red fluorescent protein; PI, propidium iodide; sfGFP, super-folding green fluorescent protein; Tae1, type VI amidase effector 1; Tse2, type VI effector 2; VipA, ClpV-interacting protein A. (TIF) [file pbio.3000720.s005.tif]

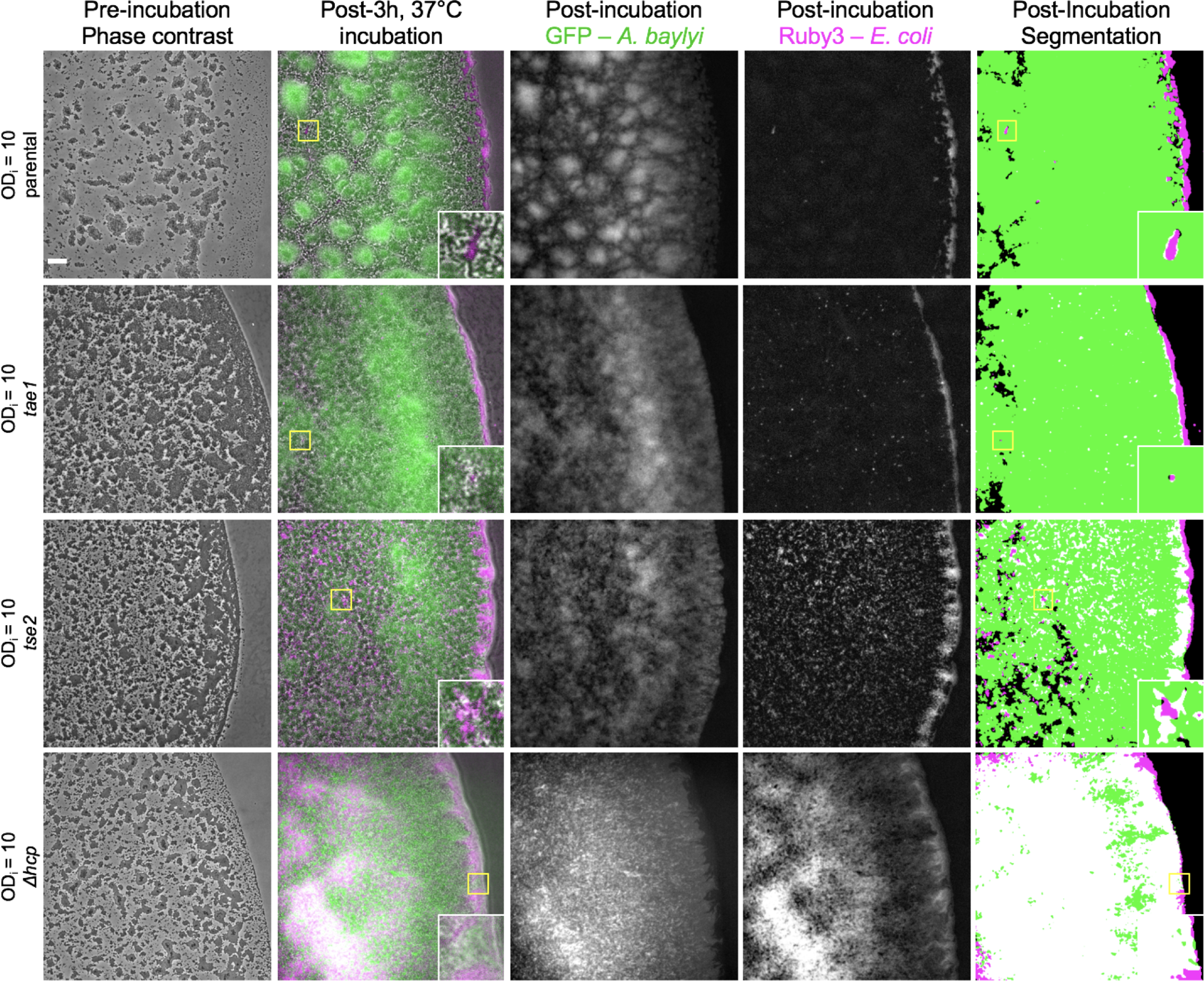

Supplement: S6 Fig — Surface competition assays comparing performance of T6SS+ attacker A. baylyi (vipA-sfGFP, green) armed with lytic (Tae1) and nonlytic (Tse2) effectors, competing with susceptible E. coli (mRuby3, magenta). Representative phase-contrast and fluorescence micrographs show pre- and post-3-hour, 37°C coincubation distributions of A. baylyi and E. coli cells, for mixtures of parental A. baylyi, Tae1, and Tse2 single-effector strains and T6SS− Δhcp mutant. Fluorescence signal for each channel was blurred and background subtracted prior to Otsu segmentation (rightmost column). Based on this, the respective area occupancies of A. baylyi and E. coli within the community were quantified; these data are plotted in Fig 2G. White pixels indicate overlapping signals for both attacker and susceptible strain. Scale bar = 50 μm; inserts depict a 50 × 50 μm field of view. Hcp, hemolysin-coregulated protein; mRuby3, monomeric red fluorescent protein PI, propidium iodide; sfGFP, super-folding green fluorescent protein; Tae1, type VI amidase effector 1; Tse2, type VI effector 2; VipA, ClpV-interacting protein A. (TIF) [file pbio.3000720.s006.tif]

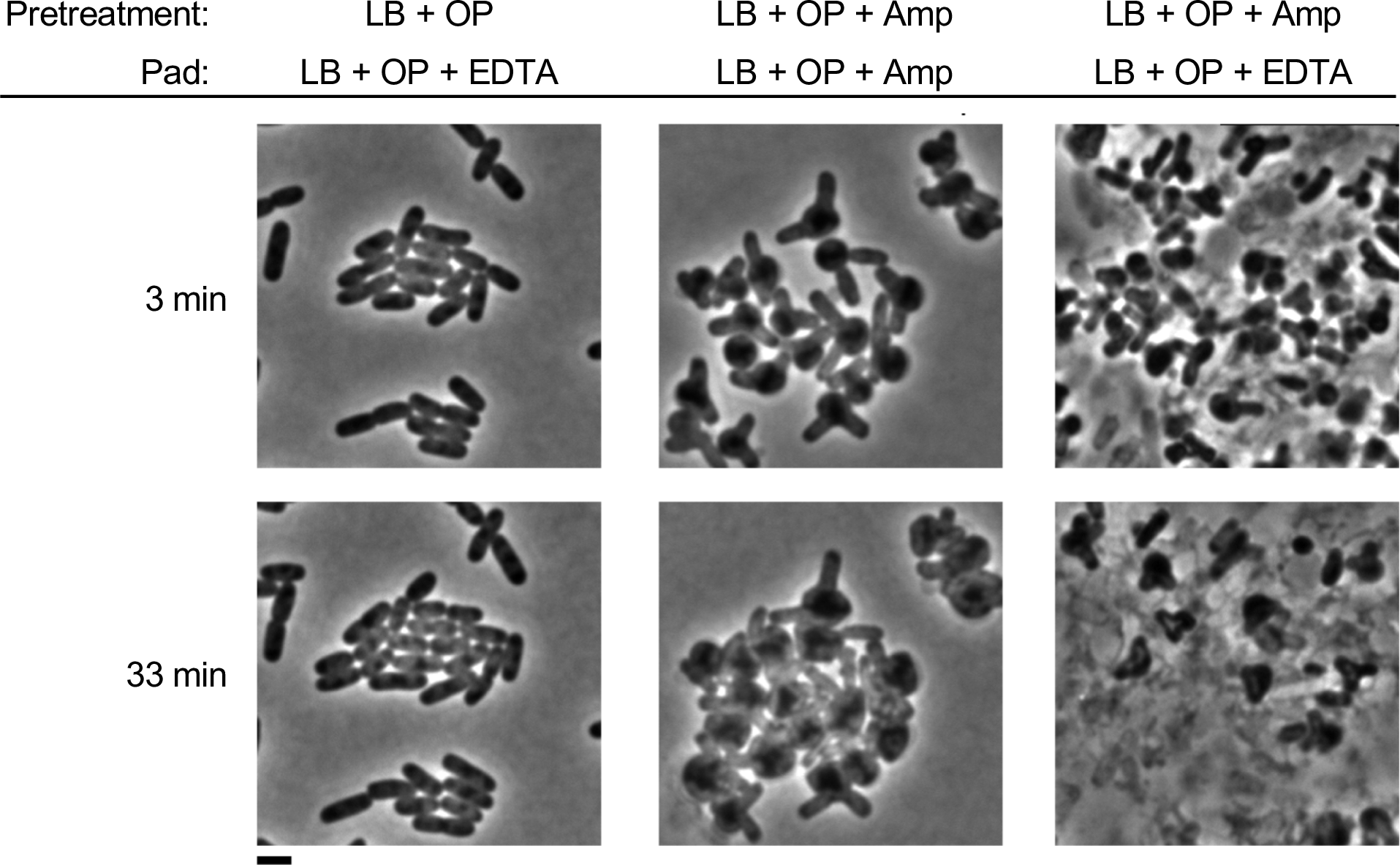

Supplement: S7 Fig — Micrographs show E. coli cells following 3-minute (top row) and 33-minute incubation (bottom row) on agarose pads, following different pretreatment conditions (column labels). OP prevents cell lysis from cell wall damage sustained from Amp (middle column); addition of 20 mM EDTA to pad media (LB) negates osmoprotection, resulting in mass lysis (right column). Scale bar: 2 μm. Amp, ampicillin; LB, lysogeny broth; OP, osmoprotectant. (TIF) [file pbio.3000720.s007.tif]

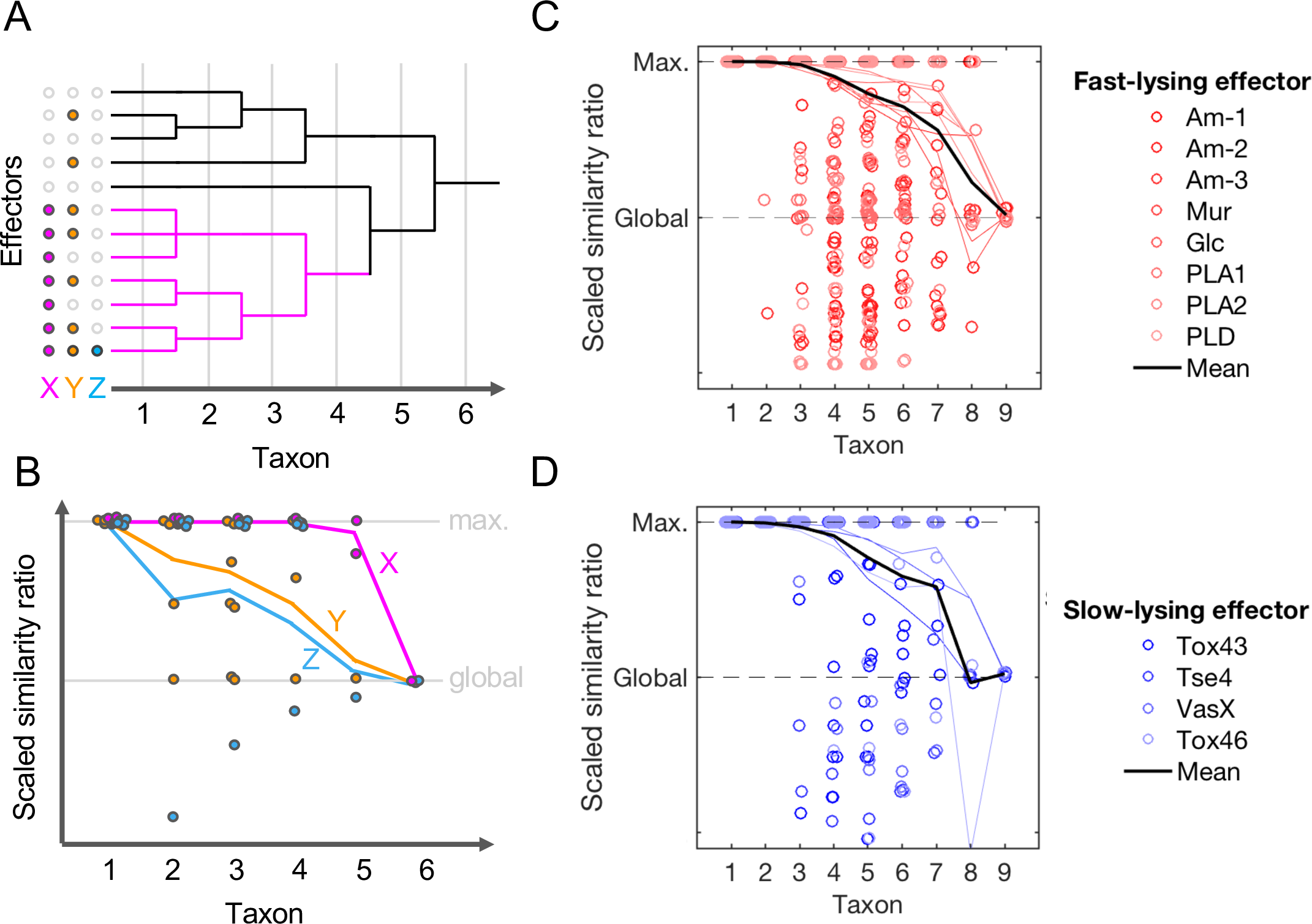

Supplement: S8 Fig — (A) Example taxonomy annotated with 3 effectors: “X,” common only to magenta clade, corresponding to strong phylogenetic signal; “Y,” randomly distributed across tree, weak phylogenetic signal; “Z,” rare effector found only in only 1 species. (B) Plot of similarity ratio (within-clade similarity normalized by tree similarity, Sce/STreee, see Materials and methods) versus taxon for the 2 effectors shown in (A). Individual points mark similarity ratios within a given clade; lines correspond to mean similarity ratio. (C) Plot of similarity ratios, analogous to (B), for the fast-lysing effectors shown in Fig 4. Values are scaled such that all effectors share the same maximum (“Max.” = 1/STreee) and converge to the same similarity ratio (“Global” = 1). Horizontal axis labels denote the following taxa: 1 = subspecies, 2 = species, 3 = subgenus, 4 = genus, 5 = family, 6 = order, 7 = class, 8 = phylum, 9 = kingdom (full tree). (D) Plot of similarity ratios, analogous to that shown in (C), for the slow-lysing effectors shown in Fig 4. Raw data are available from dx.doi.org/10.6084/m9.figshare.11980491. T6SS, type VI secretion system. (TIF) [file pbio.3000720.s008.tif]
