# Supplementary material for: The evolution of the type VI secretion system as a disintegration weapon
Source: PLoS Biol. 2020 May 26;18(5):e3000720. doi: 10.1371/journal.pbio.3000720 (PMC7274471; doi:10.1371/journal.pbio.3000720)
Supplement: Attachment — Submitted filename: Smith2019_PLOSBiol_ResponseToReviewers.docx [file pbio.3000720.s020.docx]

**Responses to Reviewers’ comments**

In this letter, we list our responses in roman text; original comments are appended for reference in italic. Our listings of specific locations, figures and tables in the manuscript correspond to those in the revised submission, except where otherwise indicated.

Reviewer #1: Robert M. Cooper, signed review

*This manuscript describes a significant and novel insight into the ecological significance and strategy of bacterial combat using the Type VI Secretion System (T6SS). In particular, injected toxins that cause rapid, catastrophic lysis of the victim cell are more effective weapons than toxins that result in a less violent demise. This is because if the victims remain intact, their remains form a "corpse layer" that shields the rest of their colony from the T6SS attackers. The ecological function of the T6SS remains relatively unexplored, and this paper represents a significant advance on understanding its function in microbial communities. The manuscript supports this interpretation using an elegant series of experiments, simulations, and analysis. These include agent-based simulations, bulk competitions on agar plates, microscopic observations in microfluidic chips, and a phylogenetic analysis of T6SS toxins. A set of experiments using osmoprotectants to prevent lysis by an otherwise lytic toxin was particularly clever, as it demonstrated that the combat advantage of lytic toxins is directly due to lysis.*

Thank you for this positive summary of our work.

*One set of experiments that would significantly bolster the authors' interpretation would be to repeat the microfluidic experiments with A. baylyi strains containing only its other lytic T6SS effector Tle1 and its other nonlytic effector Tse1. This would experimentally demonstrate generalizability of the effect and result in a stronger paper, particularly if these effectors have different killing rates from those already used, thus sampling more than just two points of the parameter space. However, if these strains are not on hand or the experiments would require a significant amount of time, the paper would still stand without them.*

We did consider adding more effectors to the original experimental design of the study, but decided against it. The reason was that this would still not control well for other differences between the effectors, most notably differences in potency (the number of effector units required to kill a target cell). Therefore, we instead chose to develop a second assay, using an osmo-protectant, that solely varied the amount of lysis caused by an effector without the confound of differences in potency or other factors. This alternative way of changing the lytic potential of effectors also demonstrated the importance of lysis for T6SS killing. With this in place, we were confident that lysis, as predicted by the simulations, is indeed important for the functioning of the T6SS whenever there are corpse barriers. From there, we then moved to the genomics study, where we again found that our predictions were also supported. Given these multiple lines of evidence to support the modelling, therefore, we have not added additional effector experiments. We hope this seems reasonable.

*Significant points:*

*1. Line 456: Killing dependence on interfacial saturation is given as k_kill=f_interface * k_fire * p_hit. However, f_interface is defined elsewhere as the fraction of the interface covered by dead cells, and increasing f_interface should decrease k_kill by providing a barrier layer. So should the dependence actually be (1-f_interface)?*

Thank you, yes. The equation should indeed read (1-f_interface); this has now been corrected in the manuscript (lines 505-506).

*2. Figure 2a,b using death stains to show a dead corpse layer for the non-lytic toxin are very important to the interpretation, but 2a seems to have significant cell death in the interior of the E. coli patch. Is this because A. baylyi have pushed inwards past dead E. coli, creating an overlap between the predator (green) and dead prey (magenta) signals? If so, it would be helpful to show the green channel by itself, as done for the magenta channel. If not, that would imply some degree of nonlocal killing, which would present a problem for the corpse barrier interpretation. If the death stain is spreading into the interior because DNA released by lysis is migrating inward, including a movie for these death stain experiments would be useful (again showing each channel separately as well as the merge).*

We think that death stain is spreading into the interior because the DNA released by lysis is migrating inward, but we agree that Fig. 2 currently leaves this ambiguous. To clarify, we have added a new SI video (SI Movie S5) that includes both *A. baylyi* and propidium iodide (PI) signals. This shows that the PI stain diffuses from the *A. baylyi* – *E. coli* interface in *both* directions. That is, it goes in towards the centre of the *E. coli* group, but also in the opposite direction back into the *A. baylyi* group:

The presence of PI in the centre of the *E. coli* cell group, therefore, is best explained by the diffusion of extracellular DNA, rather than to non-local killing or the incursion of *A. baylyi* cells into the *E. coli* group (neither of which would predict the presence of the PI stain within *A. baylyi* groups).

*3. I was confused by the description of normalized peak kill rates. The caption for Figure S2 (line 48) says "the maxima of these traces… are normalised by the number of boundary cells at each corresponding time point", but the inset to Fig. S2d shows time course data, so it must be more than just the maximum rates that were normalized. Were all time points of a given trace normalized by the number of boundary cells at the time of the maximum, or was each time point normalized by its own number of boundary cells? This could also be clarified in Methods (line 449) by specifying in the equation at what time point N_boundary is calculated.*

We apologize for this ambiguity. The procedure for this calculation is as follows:

1. For each simulation, compute the T6SS- cell death rate by calculating the gradient of the cumulative T6SS- cell kill count;
2. Take the maximum of this trace (max kill rate, which coincides with the colony reaching confluency), noting the time at which this occurs;
3. Normalize this value by the number of boundary cells (T6SS+ cells touching at least one non-T6SS+ cell) at that time.

We have reworded the text (lines 489-501) to state our methodology here more clearly.

*Minor points:*

*1. The discussion of optimal T6SS firing rates due to a tradeoff between energy costs vs killing victims is specific to unregulated T6SS firing, unlike in e.g. Pseudomonas aeruginosa, which only counter-attacks after being attacked first and directionally concentrates its fire on the aggressor, thus conserving resources. It would be good to add this caveat to the interpretation.*

This is a good point – we have assumed in our modelling that T6SS firing is constitutive. While this is realistic for the case of *A. baylyi* under our experimental conditions, other bacteria are known to vary their T6SS activity according to environmental and biotic stimuli. We have added this caveat and some supporting citations to the initial model description (lines 86-89) and to the Methodology (line 383).

*2. Coordination number is referenced but never defined.*

Apologies – by coordination number, we mean the number of neighbor cells in direct contact with a focal cell (Fig. S2b). This is now stated in the Methodology (lines 467-468).

*3. Line 237, Figure 3c caption: "toxin translocation rates" would more accurately be "kill rates", as this is what was directly measured and does not rely on an assumption about the number of required hits.*

We agree; corrected.

*Format, etc:*

*1. Line 162: extra "strain".* Corrected.

*2. Line 514: missing verb (occupancy was calculated).* Corrected.

*3. Line 538: missing "and".* Corrected.

*4. Data and code availability: still has placeholder XXX's.* We have replaced these with our reserved FigShare repository DOI (dx.doi.org/10.6084/m9.figshare.11980491).

*5. I was unable to open Movie S1 using Quicktime Player, although this opened the other movies, and I was able to open Movie S1 using VLC. The authors may wish to use a more widely compatible codec for Movie S1*. Apologies – this video does indeed have a different codec, preventing playback on some devices. We have remade it with a new codec.

Reviewer #2

*This is an excellent paper examining the role of toxin type in the evolutionary advantage of the T6SS. While a great deal is known about the mechanism and regulation of the T6SS, little is known about the factors driving its evolution. This paper provides a really interesting insight into this evolution, showing that lytic toxins delivered by the T6SS make it a more potent weapon as dead but non-lysed cells form a barrier preventing further attack of enemy strains. The authors then go on to show that lytic toxins are common across the T6SS wielding bacteria. The methods used by the authors in their simulations and experiments are state of the art, providing good mechanistic insight into how the "corpse barrier effect" operates. The quantitative agreement between the simulation models and experiments is particularly striking, providing further evidence that the corpse barrier effect is the driver of their experimental results. The manuscript is also written very clearly and the discussion of the results well balanced. I think this will make a great addition to the literature and plos biology would make a great home for it.*

Thank you for such a positive assessment.

*I have only two suggestions to improve the manuscript.*

*Firstly, while the authors experiments are elegant and careful in design, it is important to note that only one lytic and one non-lytic toxin were compared. This is understandable given the detailed nature of the authors experiments, and I am not suggesting that they carry out further experiments with additional toxins as this would be beyond the scope of the manuscript, and they have supported their predictions using comparative phylogenetics. However, I think it should be somewhat caveated that their results are for a single pair of toxins in the manuscript. This in itself provides rationale for their phylogenetic analysis, so should be easy to integrate into the manuscript.*

We agree that adding extra strains to our analyses is beyond the scope of our study and we thank Reviewer #2 for their understanding in this matter. We now highlight this limitation in the main text (lines 272-277).

*Secondly, in their phylogenetic analysis the authors simply report proportions of species and effectors that show lysis. While these proportions obviously support the authors' hypothesis, there are issues with trusting such proportions in phylogenetic studies. For example, if a very large single clade of closely related species has an over-representation of lytic toxins, then this could lead to an artificially inflated estimate of the tendency for lytic toxins to evolve. While it does not look like this is the case from the authors' phylogenetic plots, it would still be best to control for phylogeny in making these estimates. The state of the art approach here would be to fit a Bayesian phylogenetic mixed model controlling for phylogeny as a random effect (can be implemented in R using MCMCglmm), and take the estimate of the proportion of species expected to have lytic toxins from this model. This would allow for an estimate of the evolutionary tendency for T6SS toxins to be lytic while removing any confounding because of the phylogenetic distribution of the species sampled.*

Thank you for the useful suggestion. Indeed, we should have explicitly considered phylogeny in the previous version of our manuscript. However, while appropriate for other studies, we believe that the MCMCglmm method is unsuitable for our purposes. The reason is that we are not testing whether factor X is a (phylogenetically controlled) predictor of factor Y in our analysis. Rather we are simply wanting to show that there are a lot of T6SS toxins across the bacteria that are lytic. This hypothesis cannot really be phrased as a glmm, since we have no fixed effects to test as inputs against the random phylogeny.

We therefore sought an alternative way to show that the predominance of lytic effectors is not due to there being, as you say, a very large single clade of closely related species with an over-representation of lytic toxins. To do this, we developed a simple test of phylogenetic signal in our annotated taxonomy (now shown as Figure S8; Methods on lines 649-675). Briefly: for each effector, we evaluate the ratio of within-clade similarity to global similarity, for successively wider clades descending the tree (taxon = genera, families, orders, etc.). Plots of this similarity ratio vs. taxon then provide a measure of phylogenetic signal: if, say, there is one or two bacterial orders with an over-representation of a particular lytic toxin, then the similarity ratio will be very high within clades corresponding to species, genera, families and orders, and then suddenly drop for lower taxa (illustrated as a cartoon in Figure S8a,b). In contrast, we find that similarity ratios decline gradually for all 12 effectors, indicating that phylogenetic signal is generally weak.

As you note, the annotated taxonomy in Fig 4 also provides a visual estimate of phylogenetic signal. Previously we had grouped the 12 effector type annotations into 5 categories, placing a marker in a given category if a focal species possessed any of that categories’ effectors. While this made the diagram more compact, it also made it harder to assess phylogenetic signal – if adjacent species had different effectors but in the same categories, they would be marked as though identical. To address this problem, we have expanded Figure 4 to show each effector individually. This makes it easy to see that phylogeny does not explain the bias: there are many evolutionary gains and losses of effectors across the bacteria. Overall then, this suggests we should reject the hypothesis that the abundances of lytic toxins is explained by there being one, or a few, large clades that evolved particular toxins and then diversified.

Reviewer #3: Martin Ackermann, signed review

*This manuscript by William Smith and colleagues focuses on the cellular dynamics of contact-dependent killing in bacterial populations. Antagonistic interactions are common between microbial strains, and a wide-spread and prominent mechanism is based on the translocation of toxic proteins from one cell to another via a molecular machinery known as type VI secretion system. While the molecular mechanisms of type VI secretion are well known and widely studied, much less is known about the functional consequences and benefits that arise from killing. Generally, the type VI system is understood as a mechanism to eliminate competitors, but how this works exactly is not well understood. We do not know how efficiently competing cells are killed by this system, and how the efficiency depends on whether cells are planktonic or surface-attached. Addressing this knowledge gap is important for at least two reasons. First, it provides a functional and ecological context to the vibrant field of research that focuses on molecular mechanisms of type VI secretion. Second, type VI secretion is receiving increasing attention in an applied context, primarily in the context of microbial interactions in host-associated microbiomes (including the gut microbiome); understanding the dynamics of type VI-mediated killing is fundamental to any such applications.*

*Willliam Smith and colleagues combine computer simulations, single-cell analysis and comparative genomics to test one main hypothesis about the dynamics of type VI-mediated killing: the hypothesis is that, in situations where microbes grow on surfaces, type VI-mediated killing is much more efficient if the target cell is lysed than if the target cell is only killed but remains physically intact. The rationale for this hypothesis is as follows: if target cells remain intact, strains that carry a type VI-secretion system become rapidly surrounded by a layer of dead but physically intact target cells, preventing the type VI-secretion carrying strain to expand further through microbial assemblies. The authors find support for this hypothesis from their simulation and from the single-cell experiments. In addition, the analysis of bacterial genomes indicates that a large fraction of bacteria with type VI secretion systems translocate proteins that lyse the target cell, rather than just killing it.*

*In my opinion, this manuscript makes an important and original contribution to our understanding of microbial interactions, a contribution that has implications for both fundamental research as well as applied purposes. Also, the combination of modeling, genetic manipulation, quantitative single-cell analysis and comparative genomics is unique and raises the bar in the field of microbial ecology and evolution.*

We are grateful for these encouraging comments – they are greatly appreciated.

*I have a few concerns and questions that I think should be addressed.*

*A first main point (which is also mentioned by the authors on lines 207ff) is that the main experimental test has a potential caveat, namely the possibility of different killing rates mediated by the lytic and non-lytic effectors. The authors do address this issue by performing experiments with an osmo-protectant. I agree that these experiments offer additional support for their hypothesis, but think that a more detailed analysis of the single-cell experiments would potentially allow for a more direct test. Can the authors directly establish that, for the non-lytic effector, producer cells (i.e., type VI-carrying cells) and live target cells become separated by a layer of dead target cells?*

This can be seen in Figure 2a: here, live *A. baylyi* and *E. coli* cells are clearly physically separated by a layer, 1-2 cells thick, of dead (PI-stained) *E. coli* cells.

*And can they show that this effect decreases the death rate of live target cells that are separated from the producer cells by this layer? I realize that this quantification might be challenging but think that it would provide strong and direct support for the main hypothesis that the authors put forward.*

We can indeed show this. We carried out further analyses of our microfluidic chamber experiments, applying a similar Methodology as for our kill rate measurements in Figure 3c. While image analysis is indeed more challenging here (unlike the sandwich assay, the cells are not perfectly confined to a single optical plane), it is possible to measure the *E. coli* cell death rate per unit *E. coli* – *A. baylyi* interface post-confluency, with death being marked by sudden increases in PI signal either a) around a lysing cell (Tae1 case) or b) within a cell (Tse2). These measurements confirm that the *E. coli* kill rate is consistently higher for Tae1 than for Tse2, in line with our predictions. These additional analyses are now included in the main text (lines 187-191) and SI (new Figure S4 and new Movie S5).

*Related to this point, visual inspection of the lower panel of Fig. 2b gave me the impression that target cells can be killed even if they are not in direct contact with producer cells but rather several cell layers away from them. The quantification proposed above would potentially resolve this issue and provide more quantitative understanding of how spatial positioning of producers and dead and live target cells determines killing rates.*

This is an important point, also raised by Reviewer #1. As discussed above, the imaging suggests that death stain is spreading into the interior because the PI-stained DNA released by lysis is migrating inward into the cells of both species (see images above). Our new SI video (Movie S5) includes both *A. baylyi* and PI signals shows this, with the PI stain diffusing from the *A. baylyi* – *E. coli* interface into the cells of both species. If the signal were caused by *E. coli* dying behind the front, one would not expect to see the signal in the *A. baylyi* cells as well.

*Smaller comments*

*In the section about the simulation, the authors often use the term "measurements" to refer to data extracted from the simulation. I think this could be potentially confusing for readers who might misunderstand this as referring to experimentally generated data.*

Agreed – throughout, we now refer to ‘calculations / calculated values’ instead.

*Fig. 1b, d: the point that fast lysis reduces the layer of dead but intact cells does not become really clear to me from these panels (the yellow arrow don't help that much). I think a quantification of the fraction of the interstrain boundary that is occupied by dead but intact cells would be more informative.*

We have increased the magnification of the zoomed sections to make the distribution of dead cells along the boundary easier to see. For quantification, please see Figure S2j: the fraction of the boundary occupied by intact cells is just 1 minus the values shown here.

*Line 115: the term "interfacial saturation" is maybe not self-explanatory. Consider using a simpler term?*

We have changed this to ‘boundary saturation’, which we think is a better description. We also noticed that we did not define this term in the caption to Figure 1; this has now been amended (lines 117-119).

*Fig. 2e, f: what experimental data was used to generate these plots? Is this data from several biological replicates? If yes, how many? What is the variation between measurements from different replicates?*

Our apologies for the lack of clarity here. The experimental data shown are direct measurements from the chamber videos shown in Movie S3 (specifically, replicate 1). Figures 2e,f correspond to one representative replicate per treatment. but others (16 replicates in total) and the associated variability between replicates are shown later, in Figure 3e. We placed this data in a separate figure to enable comparison with our osmoprotectant data, but we now realise that we did not make this clear in the text. We now refer readers to Figure 3e in the text (lines 200-201), and Figure 2 caption (lines 167-168).

*Fig. 2e, f: I think it would be important to test the hypothesis that target cells maintain higher proportions in the case of non-lytic effector proteins directly with a statistical test.*

We agree – we performed a two-way analysis of variation (ANOVA) test to compare maximum and final *E. coli* chamber occupancies, which showed that the reduction in occupancy was statistically significant in the Tae1 case (p ≤ 0.0001), but not in the Tse2 case. This information is provided in Figure 3, but (as above) we had not previously made the link between the two figures clear. We hope that the added references to Figure 3 now resolves this problem.

*Lines 218 and 220: the authors write that they find "equal amounts of … protein" and "equal numbers of cell lysis". I think it is fundamentally not possible to establish that two experimentally measured values are "equal"; we can only report that we find no evidence that they are significantly different - but the absence of significance can have many reasons, for example a high error variance. I suggest that the authors instead make a statement about how small (or large) the difference between the two measurements is, for example by giving a confidence interval for the difference between the two measurements.*

This is a good point – we have changed our wording to reflect this (lines 226-232). We have also added confidence intervals for the difference in population means to Figure 3’s caption.

*Line 273: the numbers are not clear to me (83.2% are predicted to cause lysis and 84.8% carry at least one lytic effector - why are then not 84.8% predicted to cause lysis?)*

Apologies – here, we mean that 83% (936 / 1,125) of the *effectors* found are predicted to cause lysis, whereas 85% (396 / 466) of *species* carry at least one (predicted) lytic effector. We have reworded this sentence for clarity (lines 290-291). Please note that our numbers here deviate slightly from those in our initial submission; this is because we have now excluded 8 non-Proteobacterial species from the dataset, which we had previously included in our analyses.

*In the section about the genomic analysis, the authors suggest that DNases and pore-forming toxins do not lead to cell lysis (lines 276 and 290). Why I see that this is plausible, I would be interested in seeing more specific support for this statement - is this point supported by empirical evidence? I don't know much about the lytic effect of different type VI effectors, but for example in antibiotics the link between mode of action and cell lysis is not always obvious.*

These are good questions. This raised several issues for us that we agree would benefit from further discussion in the manuscript.

First, we now recognise that ‘lysis’ is a potentially ambiguous term, having a range of connotations for different readers and in different contexts. For our purposes, ‘lysis’ is the loss of a cell’s structural integrity, to the point where it ceases to be a physical barrier to the movement of encroaching T6SS+ cells. We now state this definition in the Methodology (lines 630-631).

Second, while we originally labelled our experimental effectors Tae1 and Tse2 as ‘lytic’ and ‘non-lytic’, classifying in terms of *lysis timescale* (i.e., the delay between effector translocation and loss of cell integrity) would offer a more complete picture. Of course, all dead bacteria will eventually lyse irrespective of toxin type, e.g. because of the cessation of wall / membrane maintenance. What matters here is the timescale of lysis, relative to the timescale of T6SS attack. Accordingly, we now refer to ‘fast-’ and ‘slow-lysing’ effectors, instead of ‘lytic’ and ‘non-lytic’, throughout the manuscript.

To check our assumptions on lysis timescales, we consulted the literature on patterns of bacterial cell death in response to different toxin categories:

1. **Peptidoglycan (PG)-targeting** T6SS effectors are well-known for being bacteriolytic (e.g. [here](https://www.nature.com/articles/nature10244)). In a previous [study](https://www.cell.com/cell-reports/pdf/S2211-1247(17)31816-8.pdf) focussing on *A. baylyi* / *E. coli* T6SS interactions, Tae1 generated lysis in < 5mins following intoxication.
2. **Phospholipases (PL)** also seem to lyse cells, but less rapidly than PG. [Here](https://www.cell.com/cell-reports/pdf/S2211-1247(17)31816-8.pdf), *A. baylyi*’s PL Tle2 was characterised as being ‘lytic’ (CPRG lysis assay); after 1h, Tle2 produced significant lysis, but only permeabilized cells on 5 min timescales. Another [study](https://www.nature.com/articles/nature12074) identified GXSXG-type T6SS PL effectors as generating lysis <15mins after intoxication, with similar patterns observed for Tle5.
3. Comparable lysis rate data are limited for T6SS **nuclease** toxins:
   a. We have been unable to find any examples of nucleases with bacteriolytic properties in the literature.
   b. We currently have a bioRxiv [preprint](https://www.biorxiv.org/content/10.1101/2020.02.25.959577v1) on colicin nuclease toxins, which shows that cells die without lysing and will sit there for several hours with an intact gfp signal, but clearly dead:

4. Lysis timescales for **pore-forming toxins** appear to depend strongly on pore size and selectivity:
   1. If the pore is non-selective (e.g. colicin [E1](https://www.nature.com/articles/276159a0) or [MACPF toxins](https://onlinelibrary.wiley.com/doi/full/10.1111/mmi.12839)), this could lead to a rapid loss of osmotic pressure, and subsequent physical collapse of the cell.
   2. However, if the pore is ion-selective, cell death occurs through membrane depolarisation rather than membrane breach. This appears to be the case for T6SS toxins like [Ssp6](https://www.nature.com/articles/s41467-019-13439-0), recently discovered in *S. Marcescens*: Ssp6 caused growth inhibition rather than cell lysis, with no detectable lysis events after 3h exposure to T6SS attackers. [Colicin N](https://onlinelibrary.wiley.com/doi/pdf/10.1111/j.1365-2958.1987.tb01938.x) behaves similarly: selective K+ efflux leads to rapid membrane depolarisation (timescale: ~2mins, see also [here](https://www.sciencedirect.com/science/article/pii/S0923250899800126)), but lysis is reported only as an after-effect only.
   3. Most pore-forming T6SS toxins appear to fall in the former category, and so are unlikely to produce lysis except as a delayed after-effect.

In summary then:

- We are defining lysis specifically as loss of cell integrity;
- Toxin-induced lysis is non-binary and best thought of as a spectrum of lysis timescales;
- Experimental measurements of lysis timescales are limited, but (so far) are broadly consistent with our prediction of Amidases and Phospholipases producing faster lysis than Nucleases & Pore-formers.

We have now added these caveats and information to the Discussion and Methods sections (lines 303-306 and 630-642).
