# Supplementary material for: The evolution of the type VI secretion system as a disintegration weapon
Source: PLoS Biol. 2020 May 26;18(5):e3000720. doi: 10.1371/journal.pbio.3000720 (PMC7274471; doi:10.1371/journal.pbio.3000720)
Supplement: Attachment — Submitted filename: Smith2019_PLOSBiol_ReResub_ResponseToReviewers.docx [file pbio.3000720.s021.docx]

**Responses to Reviewers’ comments**

In this letter, we list our responses in roman text; original comments are appended for reference in italic. Our listings of specific locations, figures and tables in the manuscript correspond to those in the revised submission, except where otherwise indicated.

Reviewer #1: Robert M. Cooper, signed review

*“The updates in this revised version address nearly all of my points, and I look forward to adding the finished paper to my library. The new Movie S5 is a useful addition that shows killing is concentrated at the species boundary.”*

We thank Reviewer #1 for assessing our revised manuscript.

*“My only remaining suggestion is to clarify the legend for Figure S2d, specifically regarding the inset. The portion I'm referring to reads: "The maxima of these traces (vertical black lines) are normalised by the number of boundary cells at each corresponding timepoint to give a kill rate per unit interface (inset), which converges to a constant value in confluent colonies." If I'm interpreting this correctly (as per the updated Methods at line 469), "maxima" should be removed when referring to the inset. The inset appears to show just the Normalized Killing Rate (NKR) with no maxima taken. The max doesn't seem to come in until the next panel S2e with normalized PEAK killing rate. So perhaps replace that sentence in the legend with something like: "These traces are normalised by the number of boundary cells at each corresponding timepoint to give a kill rate per unit interface (inset), which converges to a constant value in confluent colonies" and change the legend for E to "Normalized peak T6- kill rates taken at confluence (the maxima of the raw kill rates, vertical black lines in D), plotted against...”*

A great suggestion – now implemented (L919-927).
